# Ablation of Doublecortin-Like Kinase 1 in the Colonic Epithelium Exacerbates Dextran Sulfate Sodium-Induced Colitis

**DOI:** 10.1371/journal.pone.0134212

**Published:** 2015-08-18

**Authors:** Dongfeng Qu, Nathaniel Weygant, Randal May, Parthasarathy Chandrakesan, Mohammad Madhoun, Naushad Ali, Sripathi M. Sureban, Guangyu An, Michael J. Schlosser, Courtney W. Houchen

**Affiliations:** 1 Department of Medicine, University of Oklahoma Health Sciences Center, Oklahoma City, OK, 73104, United States of America; 2 Department of Veterans Affairs Medical Center, Oklahoma City, OK, 73104, United States of America; 3 Peggy and Charles Stephenson Oklahoma Cancer Center, Oklahoma City, OK, 73104, United States of America; 4 Department of Oncology, Beijing Chaoyang Hospital, Capitol Medicinal University, Beijing, 100020, China; 5 COARE Biotechnology, Oklahoma City, OK, 73104, United States of America; Charité, Campus Benjamin Franklin, GERMANY

## Abstract

Doublecortin-like kinase 1 (Dclk1), a microtubule-associated kinase, marks the fifth lineage of intestinal epithelial cells called tuft cells that function as tumor stem cells in *Apc* mutant models of colon cancer. In order to determine the role of Dclk1 in dextran sulfate sodium (DSS) induced colonic inflammation both intestinal epithelial specific Dclk1 deficient (Villin^Cre^;Dclk1^f/f^) and control (Dclk1^f/f^) mice were fed 3% DSS in drinking water for 9 days, allowed to recover for 2 days, and killed. The clinical and histological features of DSS-induced colitis were scored and immunohistochemical, gene expression, pro-inflammatory cytokines/chemokines, and immunoblotting analyses were used to examine epithelial barrier integrity, inflammation, and stem and tuft cell features. In DSS-induced colitis, Villin^Cre^;Dclk1^f/f^ mice demonstrated exacerbated injury including higher clinical colitis scores, increased epithelial barrier permeability, higher levels of pro-inflammatory cytokines and chemokines, decreased levels of Lgr5, and dysregulated Wnt/b-Catenin pathway genes. These results suggest that Dclk1 plays an important role in regulating colonic inflammatory response and colonic epithelial integrity.

## Introduction

Inflammatory bowel disease (IBD), consisting mainly of Crohn’s disease and ulcerative colitis is a chronic inflammatory disorder of the gastrointestinal tract caused by multiple factors [1–3]. Several factors are involved in the pathogenesis of IBD, including the presence of IBD susceptibility genes, altered microbial flora, excessive innate/adaptive immunity, defective autophagy, and reduced mucosal epithelial barrier defense [1–3]. IBD causes dysregulation of many pathways involved in the maintenance of intestinal barrier integrity such as proliferation, migration, differentiation, and cell death. These changes may eventually result in excessive tissue injury, inadequate regeneration, and an increased risk of developing cancer [1,3,4].

The adult intestinal epithelium and its tight junctions form an essential barrier between the organism and environmental factors including microorganisms. During homeostasis, the cells of the intestinal epithelium are replaced by Lgr5+ rapidly cycling stem cells originating from the crypt base [5,6]. When this process is disrupted it results in a loss of barrier integrity compromising the intestine’s ability to respond to injury [4,5]. Doublecortin-like kinase 1 (Dclk1) is a microtubule-associated protein that marks long-lived, quiescent epithelial tuft cells in the intestine, which originate from Lgr5+ cells [7–10]. A recent report utilizing a Dclk1-BAC lineage tracing mouse model demonstrated that *Apc* mutant Dclk1+ colon cells respond to inflammatory insult by initiating tumorigenesis [10]. Moreover, lineage tracing experiments with another novel Dclk1 mouse model (Dclk1^CreERT2^) demonstrated that Dclk1+ cells are tumor stem cells in *Apc* ^*min*^ mice and that inducible ablation of these cells results in complete and apparently non-toxic destruction of adenomas [9]. These findings suggest that DCLK1 may be an essential driver of *APC* mutant colorectal cancers.

Recently, we found that deleting Dclk1 in mouse intestinal epithelial cells results in impaired epithelial restoration following radiation injury [11], suggesting that Dclk1 plays an important role in response to intestinal injury. Colonic inflammation is one of the key factors that predisposes individuals to developing colon cancer [12]. In this report we characterize the effects of ablating Dclk1 in intestinal epithelial cells on the colonic response to dextran sulfate sodium (DSS)-induced colonic injury, a model that reproduces some features of human IBD. Our results demonstrate that deletion of Dclk1 in intestinal epithelial cells results in severe damage to the colon relative to control mice following DSS. These findings support an essential role for Dclk1 in maintaining intestinal epithelial barrier integrity and regulating the inflammatory response during injury.

## Materials and Methods

### Ethics Statement

All animal experiments were performed with the approval and authorization from the Institutional Review Board and the Institutional Animal Care and Use Committee, University of Oklahoma Health Sciences Center. Mice were housed under controlled conditions, including a 12-h light-dark cycle, with ad libitum access to food and water.

### Experimental animals

The *Dclk1tm1*.*2Jgg*/J mouse (Dclk1^f/f^) and the B6.SJL-Tg(Vil-cre)997Gum/J mouse (Villin^Cre^) were purchased from The Jackson Laboratory (Bar Harbor, Maine). The Villin^Cre^;Dclk1^f/f^ mouse line was generated as described previously [11]. Seven to eight-wk-old male Villin^Cre^;Dclk1^f/f^ and Dclk1^f/f^ mice were used in the experiments.

### DSS treatment

To induce colitis, both Villin^Cre^;Dclk1^f/f^ and Dclk1^f/f^ mice (n = 8 for each group) were fed with dextran sulfate sodium (DSS, molecular weight, 36,000–50,000; MP Biomedicals, Solon, OH) in drinking water (3%, wt/vol) for 9 days followed by drinking water alone for 2 days. Mice were weighed daily and scored for colitis-associated symptoms, including stool consistency (0 being a normal stool – 4 being diarrhea) and presence of fecal blood (0 being no blood in the feces, 1 being some blood in the feces, and 2 being bloody feces). At day 11, mice were killed by CO_2_ asphyxiation and colons were removed for further studies. The DSS treatment was repeated twice with n = 5 for each group of mice.

### Intestinal barrier integrity

Both Villin^Cre^;Dclk1^f/f^ and Dclk1^f/f^ mice (n = 3) were fed with 200 μl of FITC-dextran at 600 mg/kg body weight (molecular weight, 4 kDa; Sigma-Aldrich) by gavage. Mice were killed 4 hours later by CO_2_ asphyxiation and blood was collected by heart puncture. The serum concentration of the FITC-dextran (excitation, 490 nm; emission, 530 nm) was determined using a Synergy fluorometer plate reader (BioTek).

### Immunohistochemistry

Heat-induced epitope retrieval was performed on 4-μm formalin-fixed paraffin-embedded sections by utilizing a pressurized Decloaking Chamber (Biocare Medical, Concord, CA) in citrate buffer (pH 6.0) at 99°C for 18 min. For brightfield microscopy, slides were exposed to peroxidase blocking solution prior to the addition of primary antibodies (anti-Dclk1 pAb ab31704, anti-Lgr5 pAb ab137484, anti-Bmi1 pAb ab85688, anti-beta-Catenin pAb ab6302, anti-NF-κB p65 pAb ab16502, and anti-Ki67 mAb ab16667 [Abcam, Cambridge, MA]). After incubation with primary antibody overnight at 4°C, the slides were incubated in peroxidase-conjugated polymer (Promark Series-Biocare Medical, CA). Slides were developed with either Betazoid DAB or Bajoran Purple HRP chromogens (Biocare Medical). Slides were examined with a Nikon 80i microscope and DXM1200C camera for brightfield microscopy. To detect apoptotic cells, ApopTag Peroxidase in Situ Apoptosis Detection Kit was used following the manufacturer’s instructions (Millipore, Billerica, MA). The apoptotic cells were detected with anti-digoxigenin conjugated with FITC. Fluorescent images were taken with PlanFluoro objectives, utilizing a CoolSnap ES2 camera (Photometrics, Tucson, AZ). Images were processed using NIS-Elements software (Nikon Instruments, Melville, NY).

### Real-time RT-PCR Analyses

Total RNA isolated from colon was subjected to reverse transcription using Superscript II RNase H-Reverse Transcriptase and random hexanucleotide primers (Invitrogen, Carlsbad, CA). The complementary DNA (cDNA) was subsequently used to perform real-time polymerase chain reaction (PCR) by SYBR chemistry (SYBR Green I, Molecular Probes, Eugene, OR) for specific transcripts using gene-specific primers and JumpStart Taq DNA polymerase (Sigma-Aldrich, St. Louis, MO). The crossing threshold value assessed by real-time PCR was noted for the transcripts and normalized with β-actin messenger RNA (mRNA). Quantitative changes in mRNA were expressed as fold-change relative to control ± SEM value. The primer sequences for gene analyzed are listed in Table 1.

**Table 1:** Primer sequences for genes analyzed by real-time RT-PCR.

Gene ID	Forward (5’-3’)	Reverse (5’-3’)
Actb	GCTGATCCACATCTGCTGGAA	ATCATTGCTCCTCCTCAGGG
Dclk1	CAGCAACCAGGAATGTATTGGA	CTCAACTCGGAATCGGAAGACT
Ptgs2	GTTCATCCCTGACCCCCAAG	TTTAAGTCCACTCCATGGCCC
IL-6	GATGCTACCAAACTGGATATAATC	GGTCCTTAGCCACTCCTTCTGTG
IL-1β	TCGCTCAGGGTCACAAGAAA	CATCAGAGGCAAGGAGGAAAAC
Cxcl1	GCCAATGAGCTGCGCTGTTCAGTG	CTTGGGGACACCTTTTAGCATCTT
Cxcl2	GGCAAGGCTAACTGACCTGGAAAGG	ACAGCGAGGCACATCAGGTACGA
Ptgs1	CACTGGTGGATGCCTTCTCT	TCTCGGGACTCCTTGATGAC
Bmi1	GCCACTACCATAATAGAATGTCT	TTGTGAACCTGGACATCACAAA
Lgr5	CTGGGGCTCTCGGAGCTGCC	CGATGTAGGAGACTGGCGGGT

### Western Blot Analysis

Total proteins isolated from colon were subjected to Western blot analysis. The concentration of total proteins was determined by BCA protein assay (Pierce, Rockford, IL). 40 micrograms of total proteins were separated in a 7.5–15% SDS polyacrylamide gel and transferred onto a nitrocellulose membrane with a semidry transfer apparatus (Amersham Pharmacia, Piscataway, NJ). The membrane was blocked in 5% non-fat dry milk for 1 hour and probed overnight with a primary antibody. Subsequently the membrane was incubated with horseradish peroxidase-conjugated secondary antibody for 1 hour at room temperature. The proteins were detected using ECL Western blotting detection reagents (Amersham-Pharmacia). Actin (43-kD) was used as loading control and identified using a goat polyclonal IgG (Santa Cruz Biotechnology Inc., Dallas, Texas). The following antibodies were used: Dclk1 (ab31704) (Abcam, Cambridge, MA), Claudin-1 (sc-137121), Claudin-5 (sc-28670), Claudin-7 (sc-17670) (Santa Cruz Biotechnology, Dallas, TX), E-Cadherin (MA5-15711) (Life Technologies, Carlsbad, CA), β-actin (4970), NF-κB p65 (D14E12) (Cell Signaling, Danvers, MA).

### MPO activity Assay

Myeloperoxidase (MPO) activity, an index of neutrophil recruitment, was measured in colon tissue homogenates by a modification of the method of Grisham [13]. Briefly, colon homogenate was sonicated in 1% hexadecyltrimethyl-ammonium bromide buffer and subjected to centrifugation at 12,000 RPM at 4°C for 20 min. MPO activity in the homogenates was measured in triplicate using a Fluoro MPO detection kit (Cell Technology, Inc., CA) according to the manufacturer’s instruction.

### Measurement of colonic cytokines/chemokines

Colons from both Villin^Cre^;Dclk1^flox/flox^ and Dclk1^flox/flox^ mice were isolated, and homogenized in phosphate-buffered saline (PBS). Protein concentration was determined using BCA assay (Thermo Scientific, Rockford, IL). The cytokine/chemokine concentration in the colonic homogenates was measured in duplicates using a Bio-Plex Pro Mouse Cytokine 23-Plex Assay kit (Bio-Rad, Hercules, CA) according to the manufacturer’s instruction.

### Measurement of Wnt/b-Catenin pathway genes

The expression levels of mouse Wnt/b-Catenin regulated genes in colonic tissues were determined using Mouse Wnt/b-Catenin Regulated cDNA Plate Array (Signosis, Menlo Park, CA) following the manufacturer’s instructions.

### Statistical analysis

All experiments were performed in triplicate. Results were reported as average+/- SEM. Data was analyzed using the Student’s t test for comparison of mean values between groups. A *P value <*0.05 was considered statistically significant.

## Results

### Characterization of intestinal epithelial deletion of Dclk1 under normal conditions

**Fig 1:**
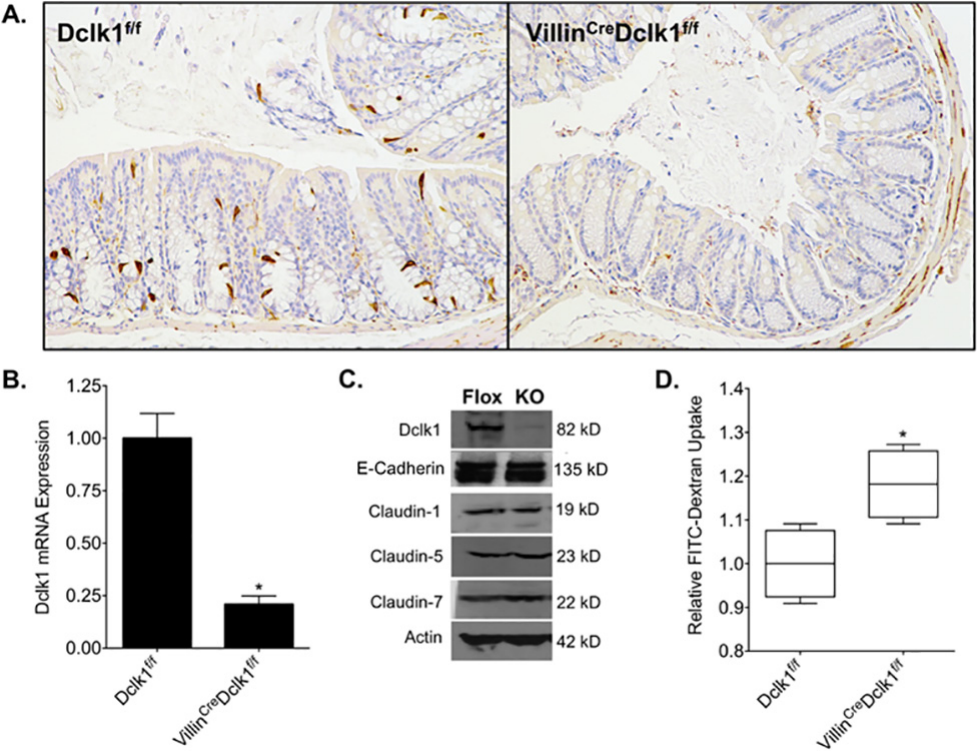
Characterization of intestinal epithelial deletion of Dclk1 under normal conditions. **A.** There is no detectable Dclk1 in the colonic epithelia of Villin^Cre^;Dclk1^f/f^ mice. Colonic tissues stained with anti-Dclk1 antibody (Brown) under normal conditions (200x magnification). **B**. Dclk1 mRNA levels were dramatically decreased in the Villin^Cre^;Dclk1^f/f^ mice; **C**. The expression levels of Dclk1 and TJPs in Dclk1^f/f^ and Villin^Cre^;Dclk1^f/f^ mice; **D**. Intestinal mucosal integrity was impaired in the Villin^Cre^;Dclk1^f/f^ mice. FITC-Dextran level in the serum of each mouse was determined 4 h after gavage (n = 3 for each group, *p<0.02).

**Fig 2:**
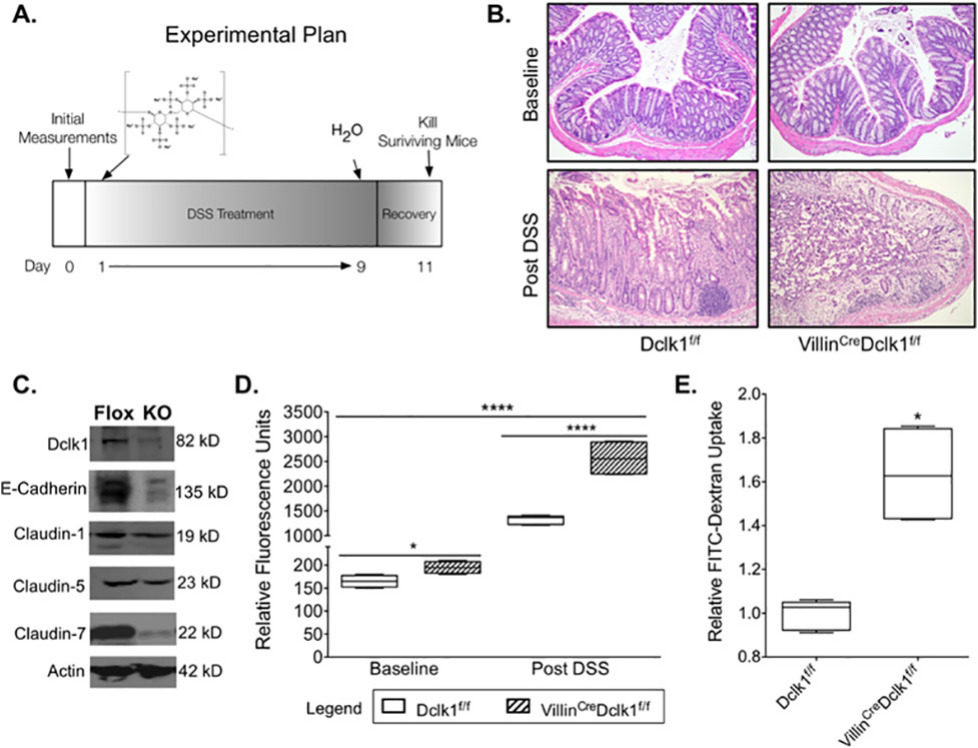
Deletion of Dclk1 exacerbates colonic barrier dysfunction following DSS treatment. **A**. Experimental plan; **B**. H&E staining demonstrated normal histology in Villin^Cre^;Dclk1^f/f^ and Dclk1^f/f^ mice at baseline and confirmed a significant inflammatory response following DSS treatment (200x magnification) **C**. Western blot analysis of tight junction and adherens junction proteins demonstrated a decrease in Claudin-1, Claudin-7, and E-cadherin in the Villin^Cre^;Dclk1^f/f^ mice after DSS treatment; **D.** FITC-Dextran levels in the serum of each mouse were determined 4 h after gavage in baseline and post DSS treatment (n = 5 for post DSS groups, *p<0.02, **** p<0.0001 Villin^Cre^;Dclk1^f/f^ vs Dclk1^f/f^); **E**. Fold change of FITC-Dextran levels in the Villin^Cre^;Dclk1^f/f^ mice post DSS treatment.

To establish a mouse line with intestinal epithelial deletion of Dclk1, we selected the Villin promoter to drive Cre-recombinase mediated excision of Dclk1 because it is specifically expressed in nearly 100% of epithelial cells of the small intestine and colon with a few notable exceptions including the brush border membrane in the proximal tubules of the kidney and glandular epithelium of the gallbladder [14]. We crossbred the constitutively active Villin^Cre^ mouse line with a mouse line with loxP sites flanking exon 3 of Dclk1 (Dclk1^f/f^), which is shared by all primary mouse isoforms [11]. Recombination in the resulting Villin^Cre^;Dclk1^f/f^ mice, resulted in a complete loss of Dclk1 protein in the colonic epithelium as demonstrated by immunohistochemistry using anti-Dclk1 antibody and this loss was also confirmed by real-time RT-PCR and Western blot (Fig 1A–1C). Despite this dramatic loss of epithelial Dclk1, hematoxylin and eosin (H&E) staining revealed no notable changes in the basal colonic architecture (Fig 2B).

The intestinal epithelium serves as an essential barrier against gut pathogens and is maintained by tight junction proteins (TJPs) [4,15]. In order to examine barrier integrity at baseline, both Villin^Cre^;Dclk1^f/f^ and Dclk1^f/f^ mice were gavaged with FITC-dextran, and serum FITC-dextran levels were determined after 4 h using a fluorescent microplate reader. Basal levels of FITC-dextran in the bloodstream were on average 20% higher in Villin^Cre^;Dclk1^f/f^ mice than in Dclk1^f/f^ mice and Western blot analysis showed a slight decrease in Claudin-1 but no other TJPs (Fig 1C and 1D). These results support our previous findings [11], and suggest that Dclk1 has a modest role in maintaining barrier function during homeostasis.

### Deletion of Dclk1 exacerbates colonic barrier dysfunction following DSS treatment

DSS treatment in mice results in inflammation and colitis consistent with human IBD [16]. To determine the effect of ablating Dclk1 in colonic epithelial cells on the response to inflammatory injury, both Villin^Cre^;Dclk1^f/f^ and Dclk1^f/f^ control mice were given 3% DSS in drinking water for 9 days followed by 2 days of water only. All animals were killed on day 11 (Fig 2A) and H & E staining was performed to confirm successful induction of colitis in all mice from both groups (Fig 2B).

Intestinal epithelial barrier integrity loss is thought to be the first event that underlies injury and inflammation in many intestinal disorders, including IBD [17,18]. To investigate epithelial Dclk1’s role in maintaining intestinal barrier integrity following DSS treatment, both Villin^Cre^;Dclk1^f/f^ and Dclk1^f/f^ mice were gavaged with FITC-Dextran before being killed, and serum FITC-dextran levels were determined after 4 hours using a fluorescent microplate reader. FITC-dextran levels in the bloodstream were dramatically increased in both groups of mice relative to baseline (Fig 2D), and were significantly higher in DSS-treated Villin^Cre^;Dclk1^f/f^ mice relative to DSS-treated Dclk1^f/f^ mice (Fig 2D and 2E). Moreover, Western blot analysis indicated significant decreases in TJPs Claudin-7 and E-cadherin, and moderate decreases in Claudin-1, -5 (Fig 2C). These results demonstrate that loss of epithelial Dclk1 further compromises barrier integrity following inflammatory injury of the colon and support our previous findings that Dclk1 is essential to maintaining barrier integrity through TJPs following intestinal injury [11].

### Deletion of Dclk1 increases epithelium apoptosis and decreases epithelium proliferation following DSS treatment

**Fig 3:**
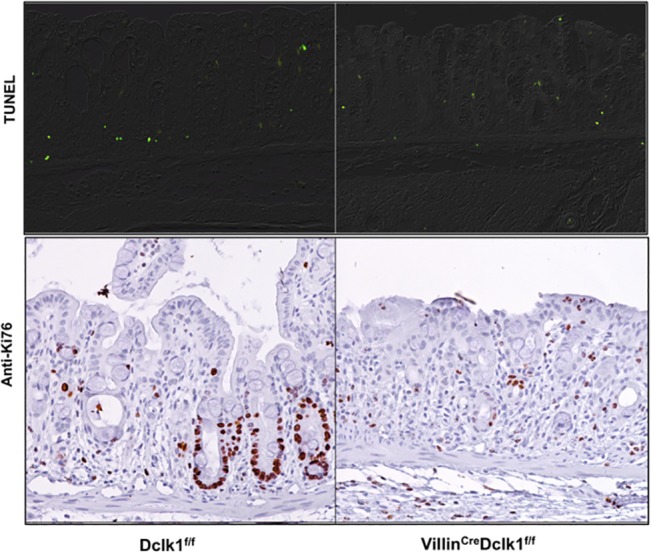
Deletion of Dclk1 increases epithelial apoptosis and decreases epithelial proliferation following DSS treatment. **A.** Apoptotic cells (Green) identified by TUNEL assay; **B.** Proliferative cells identified by immunostaining with anti-Ki67 antibody.

The colonic epithelial response to DSS injury was also assessed in terms of cell apoptosis and proliferation, which are both important factors in regeneration following injury. Apoptotic cells were identified by TUNEL assay (Fig 3), and there was a slight increase in apoptotic cells detected in Villin^Cre^;Dclk1^f/f^ relative to Dclk1^f/f^ control mice. To examine the effect of ablating Dclk1 on colonic epithelium proliferation, colonic tissues were stained with anti-Ki67 antibody (Fig 3), and proliferative colonic crypts were identified in Dclk1^f/f^ control mice, but the presence of Ki67+ proliferative cells in Villin^Cre^;Dclk1^f/f^ mice crypts was negligible. These results demonstrate that Dclk1 plays an important role in epithelial apoptosis and proliferation following injury.

### Deletion of Dclk1 exacerbates DSS-induced inflammation of the colonic mucosa

**Fig 4:**
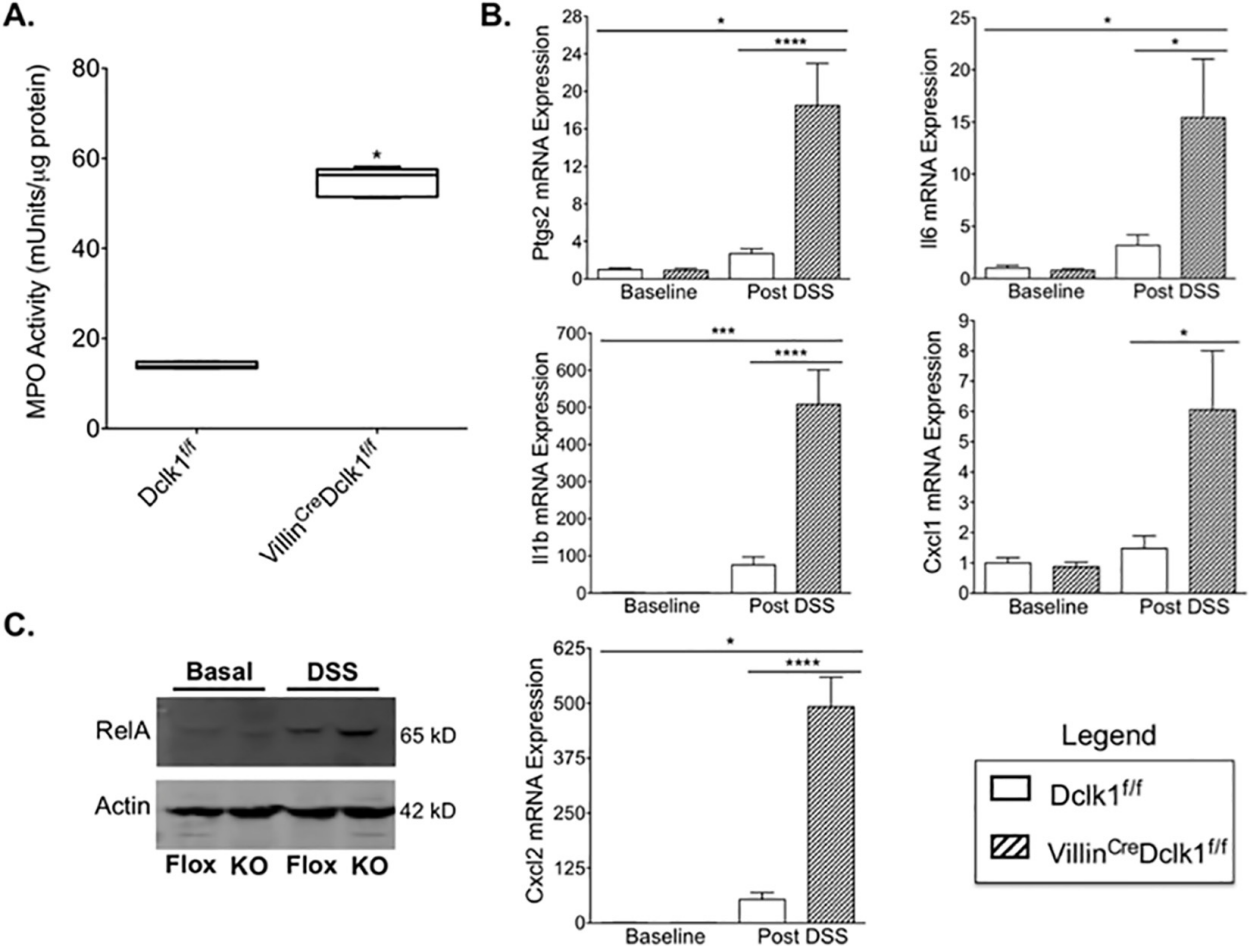
Deletion of epithelial Dclk1 exacerbates DSS-induced inflammation of the colonic mucosa. **A.** Colonic MPO activity was dramatically increased in the Villin^Cre^;Dclk1^f/f^ mice following DSS treatment; **B**. The mRNA expression levels of Ptgs2, Il6, Il1β, Cxcl1, and Cxcl2 in colonic tissues were measured by quantitative real-time RT-PCR, and normalized against β-actin. Values in the bar graphs are given as mean ± SEM, *p<0.02, **** p<0.0001 Villin^Cre^;Dclk1^f/f^ vs Dclk1^f/f^; **C**. The protein expression level of RelA in colonic tissues was analyzed by Western blotting.

**Fig 5:**
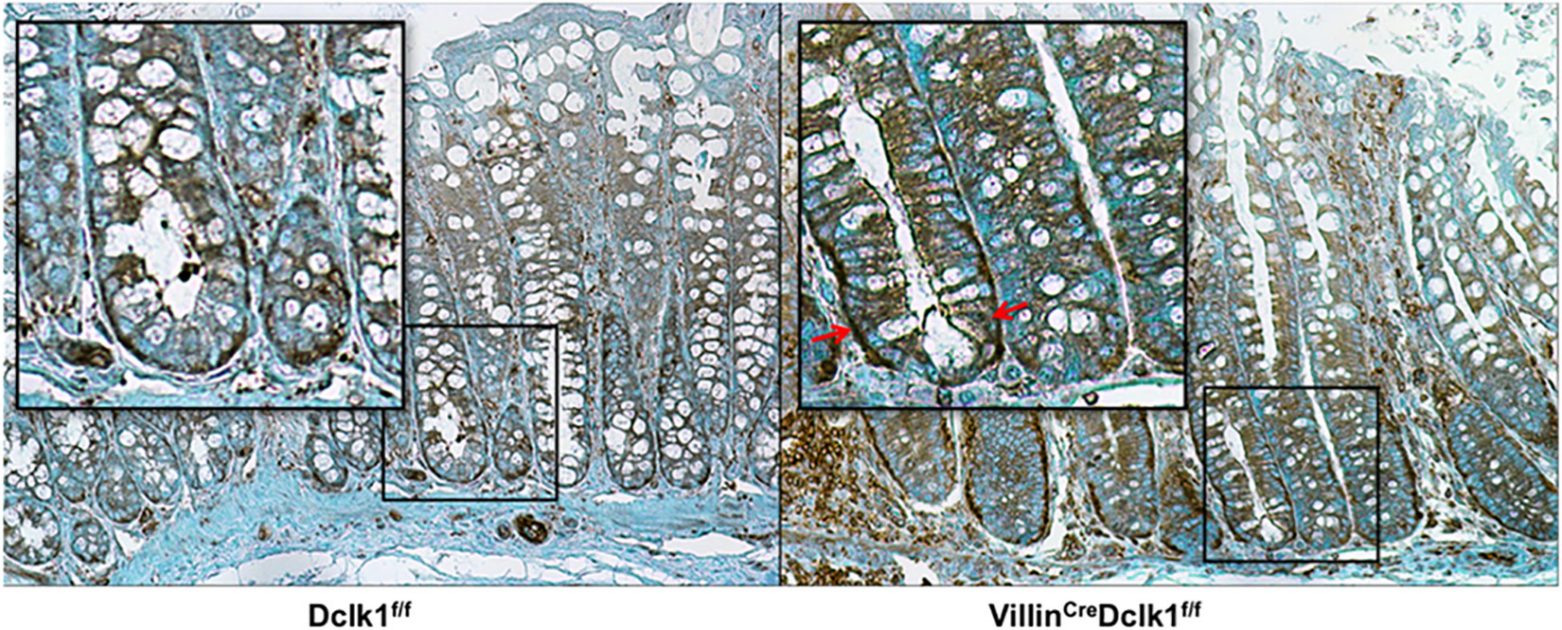
Deletion of Dclk1 increases NF-κB expression following DSS treatment. The expression of NF-κB p65 (Brown) was identified by immunostaining with anti-p65 antibody. Occasional nuclear localization of p65 was indicated by arrow in Villin^Cre^;Dclk1^f/f^ mice. Alcian blue was used for counterstaining.

In the gut, IBD is characterized by extensive neutrophil infiltration [19], which is related to the severity of inflammation. In order to determine the level of neutrophil infiltration we quantified myeloperoxidase (MPO) activity in the colon tissues of DSS-treated mice. Following DSS, MPO activity was more than 3 fold higher in Villin^Cre^;Dclk1^f/f^ mice compared to Dclk1^f/f^ mice (Fig 4A), suggesting a dramatic increase in neutrophil infiltration in intestinal epithelial Dclk1 deficient mice. Pro-inflammatory cytokines and chemokines produced by activated neutrophils play an important role in mediating tissue inflammation and injury, and prostaglandin-endoperoxide synthase 2 (Ptgs2/Cox2) is selectively induced by proinflammatory cytokines at the site of inflammation [20]. Ptgs2 mRNA levels were approximately 6 fold higher in the colon tissue of Villin^Cre^;Dclk1^f/f^ mice relative to Dclk1^f/f^ after DSS treatment (Fig 4B). Consistent with this finding, the mRNA expression levels of several pro-inflammatory cytokines and chemokines including Il6, Il1β, Cxcl1, and Cxcl2 were also 4–6 fold higher in the colon of Villin^Cre^;Dclk1^f/f^ mice relative to control mice after DSS treatment (Fig 4B). Nuclear factor kappa B (NF-κB) is a family of ubiquitously expressed transcription factors that strongly influences the course of mucosal inflammation [21]. Western blot analysis demonstrated upregulation of the RelA (p65) subunit of NF-κB in the colon of both groups of mice following DSS treatment and its expression was significantly increased in DSS-treated Villin^Cre^;Dclk1^f/f^ mice relative to Dclk1^f/f^ mice (Fig 4C). In addition, colonic tissues were also stained with anti-RelA antibody, and there was a significantly increased RelA expression detected in Villin^Cre^;Dclk1^f/f^ relative to Dclk1^f/f^ control mice with occasional nuclear localization of RelA found in Villin^Cre^;Dclk1^f/f^ mice but not in Dclk1^f/f^ control mice (Fig 5).

**Fig 6:**
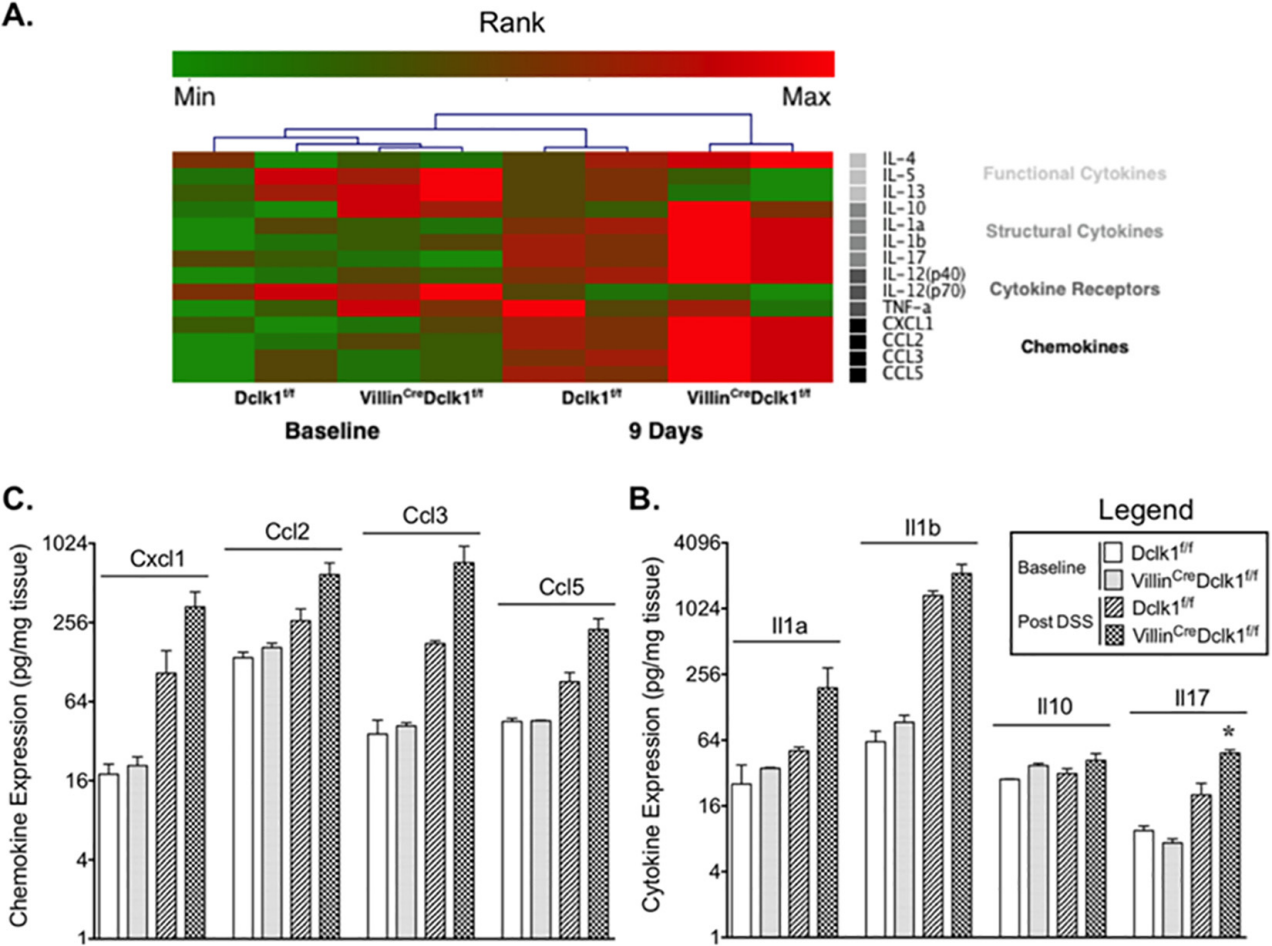
Epithelial deletion of Dclk1 upregulates pro-inflammatory cytokines and chemokines after DSS treatment. **A.** Heatmap of Bio-Plex Pro Mouse Cytokine 23-Plex Assay results. **B**. The individual expression levels of structural cytokines, IL-1α, IL-1β, IL-10, and IL17. **C**. The individual expression levels of chemokines, Cxcl1, Ccl2, Ccl3, and Ccl5.

To further investigate this phenomenon we measured the levels of 23 cytokines and chemokines in treated and untreated colon tissue of both mouse strains using a magnetic-bead based immunoassay. We found increased expression of both cytokines and chemokines in Villin^Cre^;Dclk1^f/f^ mice relative to Dclk1^f/f^ mice after DSS treatment (Fig 6A). Particularly, the proinflammatory structural cytokines IL-1α, IL-1β, and IL-17 and the proinflammatory chemokines Cxcl1, Ccl2, Ccl3, and Ccl5, were upregulated further in the Villin^Cre^;Dclk1^f/f^ mice compared to Dclk1^f/f^ mice (Fig 6B and 6C). Collectively, these results support our hypothesis that deleting Dclk1 in the intestinal epithelium increases tissue inflammation following injury. Additionally, these results suggest that Dclk1 regulates NF-κB expression, and may have a direct role in regulating the inflammatory response to DSS-induced injury.

### Deletion of Dclk1 exacerbates clinical features of colitis following DSS treatment

**Fig 7:**
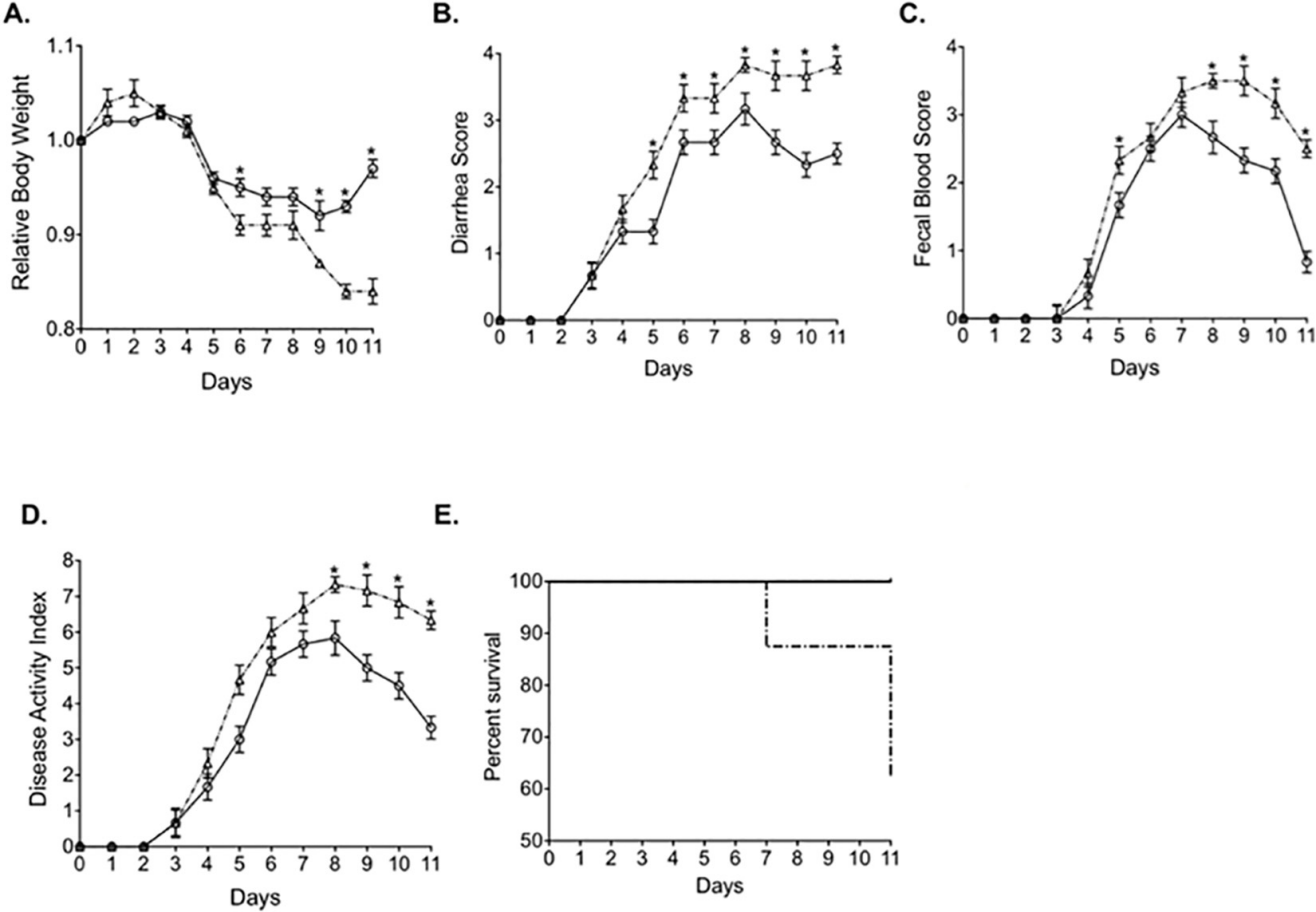
Dclk1 epithelial deletion increases disease activity following DSS-induced colitis. Both Villin^Cre^;Dclk1^f/f^ and Dclk1^f/f^ mice were fed with 3% DSS in drinking water for 9 days followed by regular drinking water for 2 days to induce colitis (n = 8 for each group). **A.** Body weight. **B**. Diarrhea score. **C**. Fecal blood score. **D**. Disease activity index. **E**. Kaplan-Meier survival curve (dashed line = Villin^Cre^;Dclk1^f/f^, solid line = Dclk1^f/f^). Data are presented as the mean ± S.E.M. *p<0.05 Villin^Cre^;Dclk1^f/f^ vs Dclk1^f/f^.

DSS-induced injury reproduces some clinical features of human colitis including weight loss, diarrhea, and bloody stools [16]. At baseline and throughout the course of treatment we tracked these features on a daily basis. As expected, the body weights in both groups of mice were decreased after DSS treatment. However, towards the end of treatment the percentage of body weight loss in Villin^Cre^;Dclk1^f/f^ mice was higher than in Dclk1^f/f^ mice (Fig 7A). Besides weight loss, diarrhea and bloody stools were observed in both groups of mice, and significantly more severe diarrhea and fecal blood resulted from DSS-treatment of Villin^Cre^;Dclk1^f/f^ mice compared to Dclk1^f/f^ mice (Fig 7B and 7C). The combination of these two scores indicated a consistently and significantly higher disease activity in Villin^Cre^;Dclk1^f/f^ mice (Fig 7D). Consequently, during the course of DSS treatment, all Dclk1^f/f^ mice survived, while approximately 35% of Villin^Cre^;Dclk1^f/f^ mice died following DSS treatment (Fig 7E). These findings demonstrate that deletion of Dclk1 in intestinal epithelium exacerbates the clinical features of DSS-induced colitis resulting in increased mortality.

### Deletion of epithelial Dclk1 alters the intestinal tuft and stem cell signature

**Fig 8:**
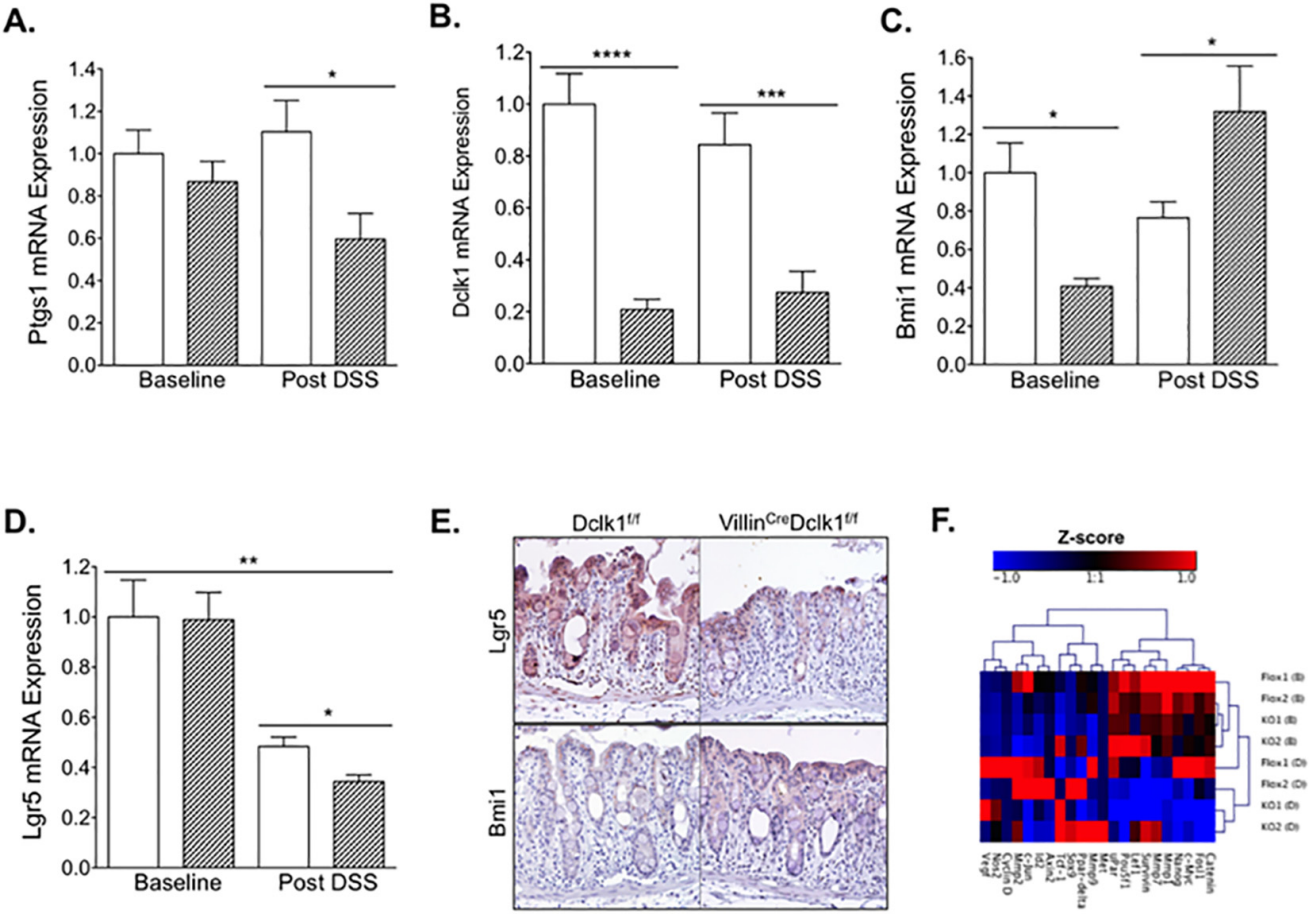
Deletion of epithelial Dclk1 alters the intestinal tuft and stem cell signature. Total RNAs were isolated from colonic tissue. The mRNA expression levels of Dclk1, Ptgs1, Lgr5, and Bmi1 (**A, B, C, D**) were measured by quantitative real-time RT-PCR, and normalized against β-actin (dashed bar = Villin^Cre^;Dclk1^f/f^, open bar = Dclk1^f/f^). Values in the bar graphs are given as mean ± SEM and *, **, ***, **** denote statistically significant differences (*p*<0.05, <0.005, <0.0005, 0.00005 respectively). **E**. Colonic tissues were stained with anti-Lgr5 and anti-Bmi1 antibodies (Brown) post DSS treatment. **F**. The expression levels of mouse Wnt/b-Catenin regulated genes in colonic tissues were determined using Mouse Wnt/b-Catenin Regulated cDNA Plate Array.

In order to determine the effect of deleting Dclk1 in intestinal epithelial cells on intestinal tuft and stem cell populations before and after DSS treatment, we measured the mRNA expression levels of signature genes from these cell populations. Ptgs1 (Cox1), a prominent tuft cell marker, was not affected by DSS treatment in Dclk1^f/f^ mice, but was decreased approximately 30% in Villin^Cre^;Dclk1^f/f^ mice following DSS treatment (Fig 8A). Although tuft cell marker Dclk1 mRNA appeared slightly decreased in Dclk1^f/f^ mice following treatment this decrease was not significant (Fig 8B).

To assess the effects of DSS treatment on both actively cycling stem cell and reserve stem cell populations, the mRNA expression levels of Lgr5 and Bmi1 were determined. The expression level of cycling stem cell marker Lgr5 was decreased more than 50%, and the expression level of Bmi1 decreased about 20% in Dclk1^f/f^ mice after DSS treatment (Fig 8C and 8D), suggesting a decrease in both stem cell populations following DSS-induced colitis. Moreover, Lgr5 expression levels were further decreased in Villin^Cre^;Dclk1^f/f^ mice compared to Dclk1^f/f^ mice after DSS treatment (Fig 8D) while Bmi1 expression levels were dramatically upregulated in Villin^Cre^;Dclk1^f/f^ mice compared to Dclk1^f/f^ mice after DSS treatment (Fig 8C). In confirmation of these findings, decreased expression of Lgr5 and increased expression of Bmi1 in distal colon tissues post DSS treatment were also observed in Villin^Cre^;Dclk1^f/f^ mice by immunostaining (Fig 8E). This may suggest activation of the Bmi1^+^ reserve stem cell population in response to the exacerbated injury present in the Villin^Cre^;Dclk1^f/f^ mice and a potential compensatory mechanism for the loss of Dclk1.

## Discussion

Intestinal barrier dysfunction is an integral feature of IBD including Crohn’s disease and ulcerative colitis. Barrier disruption in IBD compromises epithelial tight junctions due to reduced TJP expression and dysregulated subcellular localization. The consequences of these barrier-related defects are often exacerbated by increased pro-inflammatory cytokine and chemokine activity in the inflamed colon. Although the precise etiology of IBD remains unclear, chronic inflammation is associated with poor immune response resulting from genetic predispositions, altered populations of the intestinal microbiome, and deficient responses to these populations [3,15].

The DSS model is a well-characterized chemical irritant mouse model that begins with barrier disruption thereby encompassing a key feature of human IBD [16]. The resultant loss of direct and indirect regulation of genes and pathways critical to the recovery process is a key element in the response to DSS-related injury. Structurally, tuft cells are still present in Villin^Cre^;Dclk1^f/f^ knockout mice and this study again implicates Dclk1 in the maintenance of barrier integrity and as a regulator of factors affecting survival in acute severe cytotoxic colonic injury [10,11].

In this report we provide a detailed assessment of the cytokine and chemokine profiles of Villin^Cre^;Dclk1^f/f^ mice with genetically depleted intestinal and colonic Dclk1. The dramatically exaggerated chemokine and cytokine expression profile supports a critical role for Dclk1-based regulation of pro-inflammatory mediators as well as the notion that the Dclk1 regulatory balance is required for adequate restitution of injury-mediated barrier disruption. These findings are consistent with a recent report in which diphtheria-toxin driven ablation of Dclk1+ tuft cells was utilized, and demonstrate that, in terms of survival which was nearly the same in the aforementioned study and the present study, the loss of Dclk1 protein alone is comparable to the loss of the entire tuft cell [10]. Additionally this study illustrates a potential mechanistic delineation of the effects of barrier dysfunction and Dclk1-mediated regulation of the inflammatory response that was not explored in the tuft cell deletion model used by Westphalen et. al [10].

**Fig 9:**
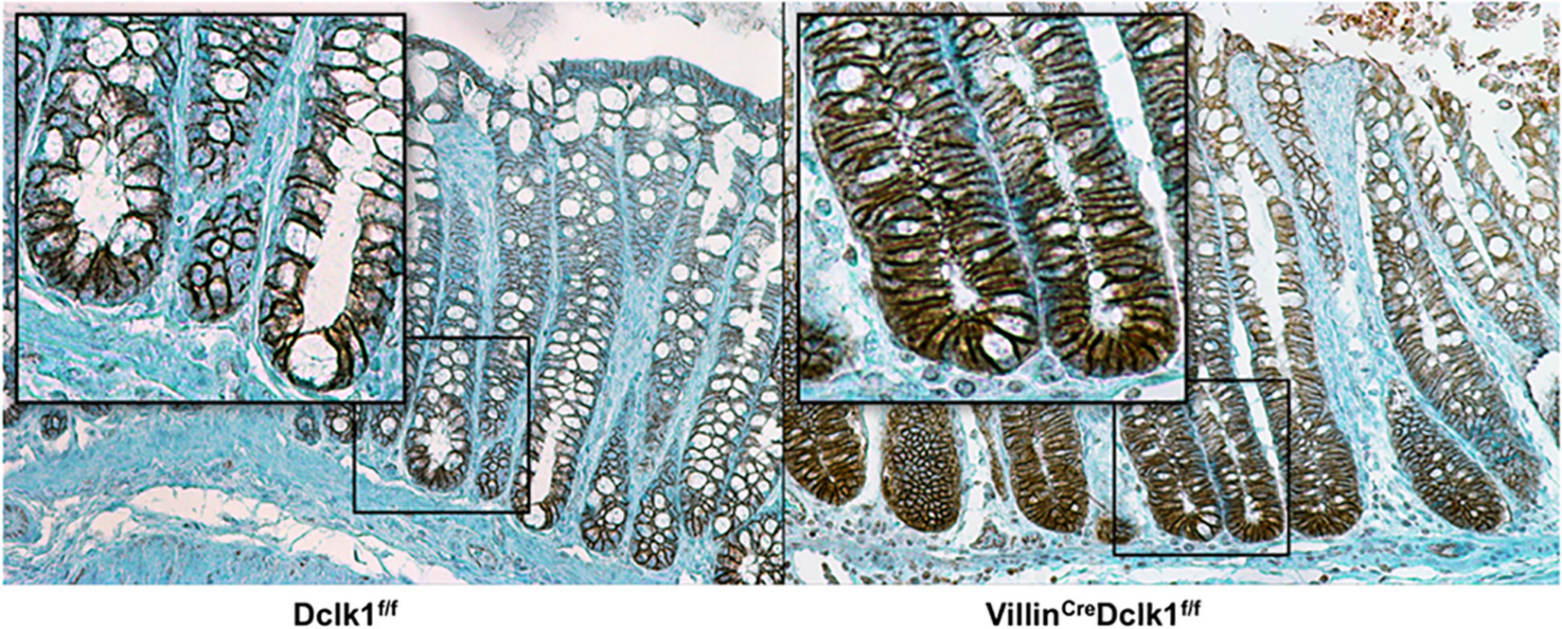
Immunohistochemical staining of β-Catenin in colon tissues following DSS treatment. The expression of β-Catenin (Brown) was identified by immunostaining with anti- β-Catenin antibody. Increased expression and increased nuclear localization of β-Catenin were found in Villin^Cre^;Dclk1^f/f^ relative to Dclk1^f/f^ mice. Alcian blue was used for counterstaining.

Although multiple lines of evidence indicate that Dclk1 is a regulator of stem cells in the intestinal niche [10,11,22], questions remain about the extent to which Dclk1 protein regulates the tuft cell’s chemosensory and secretory functions and how this affects the intestinal stem cell niche. RNA-seq analysis of the small intestinal epithelium of Villin^Cre^;Dclk1^f/f^ knockout mice demonstrates dysregulation of key chemosensory and secretory markers such as guanylin, uroguanylin, proenkephalin, proopiomelanocortin, and various taste receptors which may modulate stem cell dynamics and function [11]. In order to assess whether the loss of Lgr5 expression in Villin^Cre^;Dclk1^f/f^ mice may be related to dysregulation of Wnt/β-Catenin signaling, we used an RT-PCR array to measure expression levels of selected Wnt pathway genes. We found dysregulated expression at baseline and following DSS in Villin^Cre^;Dclk1^f/f^ mice compared to Dclk1^f/f^ mice suggesting that Dclk1 is an active regulator of Lgr5 and Wnt signaling (Fig 8F). In addition, immunohistochemical studies indicated that there is a significantly increased nuclear localization of β-Catenin in colonic crypts detected in Villin^Cre^;Dclk1^f/f^ relative to Dclk1^f/f^ control mice (Fig 9), suggesting that the WNT-β-Catenin signaling pathway is still very active in the knockout mice while the control epithelium has already regenerated. Moreover, proliferative colonic crypts, as marked by Ki67+ cells, were barely identifiable in Villin^Cre^;Dclk1^f/f^ mice following DSS treatment (Fig 3), suggesting that deletion of Dclk1 in colonic epithelium retards the β-Catenin-driven crypt regeneration process in response to severe injury somewhere downstream of β-Catenin nuclear translocation. Further WNT-centric studies will be necessary to understand this crosstalk between this important pathway, Dclk1, and the Dclk1+ tuft cell.

## Supporting Information

S1 File NC3Rs ARRIVE Guidelines Checklist. (PDF)
